# Weighted Gene Co-expression Network Analysis Identified a Novel Thirteen-Gene Signature Associated With Progression, Prognosis, and Immune Microenvironment of Colon Adenocarcinoma Patients

**DOI:** 10.3389/fgene.2021.657658

**Published:** 2021-07-12

**Authors:** Cangang Zhang, Zhe Zhao, Haibo Liu, Shukun Yao, Dongyan Zhao

**Affiliations:** ^1^Department of Pathogenic Microbiology and Immunology, School of Basic Medical Sciences, Xi’an Jiaotong University, Xi’an, China; ^2^Key Laboratory of Resource Biology and Biotechnology in Western China (Ministry of Education), College of Life Science, Northwest University, Xi’an, China; ^3^Department of Hematology, The First Affiliated Hospital of Xi’an Jiaotong University, Xi’an, China; ^4^Graduate School, Chinese Academy of Medical Sciences and Peking Union Medical College, Beijing, China; ^5^Department of Gastroenterology, China-Japan Friendship Hospital, Beijing, China

**Keywords:** colon adenocarcinoma, weighted gene co-expression network analysis, prognosis, NAT1, NAT2, immune infiltration

## Abstract

Colon adenocarcinoma (COAD) is one of the most common malignant tumors and has high migration and invasion capacity. In this study, we attempted to establish a multigene signature for predicting the prognosis of COAD patients. Weighted gene co-expression network analysis and differential gene expression analysis methods were first applied to identify differentially co-expressed genes between COAD tissues and normal tissues from the Cancer Genome Atlas (TCGA)-COAD dataset and GSE39582 dataset, and a total of 309 overlapping genes were screened out. Then, our study employed TCGA-COAD cohort as the training dataset and an independent cohort by merging the GES39582 and GSE17536 datasets as the testing dataset. After univariate and multivariate Cox regression analyses were performed for these overlapping genes and overall survival (OS) of COAD patients in the training dataset, a 13-gene signature was constructed to divide COAD patients into high- and low-risk subgroups with significantly different OS. The testing dataset exhibited the same results utilizing the same predictive signature. The area under the curve of receiver operating characteristic analysis for predicting OS in the training and testing datasets were 0.789 and 0.868, respectively, which revealed the enhanced predictive power of the signature. Multivariate Cox regression analysis further suggested that the 13-gene signature could independently predict OS. Among the 13 prognostic genes, *NAT1* and *NAT2* were downregulated with deep deletions in tumor tissues in multiple COAD cohorts and exhibited significant correlations with poorer OS based on the GEPIA database. Notably, *NAT1* and *NAT2* expression levels were positively correlated with infiltrating levels of CD8+ T cells and dendritic cells, exhibiting a foundation for further research investigating the antitumor immune roles played by *NAT1* and *NAT2* in COAD. Taken together, the results of our study showed that the 13-gene signature could efficiently predict OS and that *NAT1* and *NAT2* could function as biomarkers for prognosis and the immune response in COAD.

## Introduction

Due to a number of factors including environmental exposure to carcinogens and genetic predisposition, the morbidity and mortality rates of colorectal cancer are increasing rapidly, and more than 2.2 million new cases are expected to be diagnosed, accounting for 1.1 million cancer-related deaths by 2030 (Arnold et al., 2017; Islami et al., 2018). Colon adenocarcinoma (COAD) is the most frequently diagnosed histological subtype of colorectal cancer, ranking fourth in terms of incidence and mortality among all kinds of malignant tumors in 2018 (Bray et al., 2018). Although considerable progress has been made in the early diagnosis strategies and multidisciplinary cancer management in recent decades, the invasion, migration, metastasis and recurrence of COAD have been bottlenecks for improving the long-term survival of patients, and these bottlenecks have kept the 5-year survival rate for patients diagnosed with COAD from exceeding 30% (Siegel et al., 2017; Watanabe et al., 2018; Li et al., 2019). Conventional methods utilizing the American Joint Committee on Cancer (AJCC) tumor node metastasis (TNM) classification system, vascular invasion and other parameters are widely employed to predict prognosis and guide treatment in COAD. However, considering the high genetic heterogeneity of COAD, disease metastasis, progression and clinical outcomes cannot be accurately predicted based on conventional staging methods (Weiser et al., 2011; Cancer Genome Atlas Network, 2012; Guinney et al., 2015). Although patients suffering from COAD may be in the same TNM stage, their clinical outcomes may differ considerably. Therefore, it is highly important to identify accurate prognostic biomarkers to understand the pathogenesis, predict clinical outcomes and devise personalized therapies in COAD.

Genome-sequencing technological development has strongly affected our understanding of the molecular mechanisms of colorectal carcinogenesis, and an increasing number of scientists have recognized the considerable potential of molecular signatures at the genetic level in predicting COAD prognosis. It has been reported that single genetic alterations, such as DNA mismatch repair (MMR) genes, *BRAF*, and *KRAS*, might represent as novel markers for predicting the prognosis of COAD (Punt et al., 2017). COAD is a molecularly complex disease that develops via the inactivation of tumor suppressor genes and the activation of oncogenes, suggesting that a single prognostic biomarker may differentiate COAD patients into different prognostic subgroups less reliably than a multiparameter molecular signature (Nguyen et al., 2020). Extensive studies have been conducted to investigate multigene-based signatures for the prediction of prognosis outcomes in COAD. For example, Ge et al. (2020) established a five-gene prognostic signature (*SMAD4*, *MUC16*, *COL6A3*, *FLG*, and *LRP1B*) that discriminates patients with stage III COAD into good- and poor-prognostic subgroups. Another study constructed a six-gene signature (*EPHA6*, *TIMP1*, *IRX6*, *ART5*, *HIST3H2BB*, and *FOXD1*) that accurately identified COAD patients at high risk of death (Zuo et al., 2019). However, few of these models have been widely applied in clinical practice, and a systematic study integrating gene expression profiling data from multiple source meta-analyses and improving statistical power for differentially expressed gene (DEG) identification are highly important for constructing more accurate and reproducible prognostic models. In addition, since a growing number of studies have identified hub genes that are increased in tumors tissues as compared with normal specimens, the tumor suppressor roles played by downregulated genes in tumors have largely been overlooked (Lv and Li, 2019; Yuan et al., 2020b). It is also important to explore the molecular mechanisms underlying hub genes that exhibit weak expression in tumors and are involved in the occurrence and development of COAD.

The overall goal of this study was to evaluate gene expression changes between COAD and normal samples and identify hub genes with prognostic value in COAD. Recently, considerable gene expression information regarding multiple carcinomas has been obtained from publicly available genomic datasets, such as The Cancer Genome Atlas Cancer Genome (TCGA) and Gene Expression Omnibus (GEO), and deep mining of both datasets has good application prospects in exploring cancer biology and identifying potential biomarkers for cancer diagnosis, treatment and prognosis (Chibon, 2013). In the current study, the transcriptomic expression data of the GEO GSE39582 dataset and TCGA-COAD dataset were downloaded and subjected to DEG analysis to evaluate gene expression changes between COAD and normal samples. Weighted gene co-expression network analysis (WGCNA) was employed to screen highly correlated gene clusters with COAD tumorigenesis. WGCNA, a powerful bioinformatic method, is widely used to detect potential modules of highly correlated genes and hub genes associated with clinical features on the basis of the theory that genes with similar functions or involved in common biological regulatory pathways may have similar co-expression patterns. Furthermore, univariate and multivariate Cox regression analyses were performed to select novel prognostic genes associated with the overall survival (OS) of COAD patients among the above genes and establish a stepwise 13-gene prognostic model. The prognostic performance of the 13-gene model was characterized by using the TCGA-COAD dataset and further validated in an independent dataset by merging the GSE39582 and GSE17536 datasets. Finally, in-depth bioinformatic analyses were employed to identify the underlying regulatory mechanisms of the identified prognosis-related genes.

## Materials and Methods

### Data Sources and Processing

**FIGURE 1:**
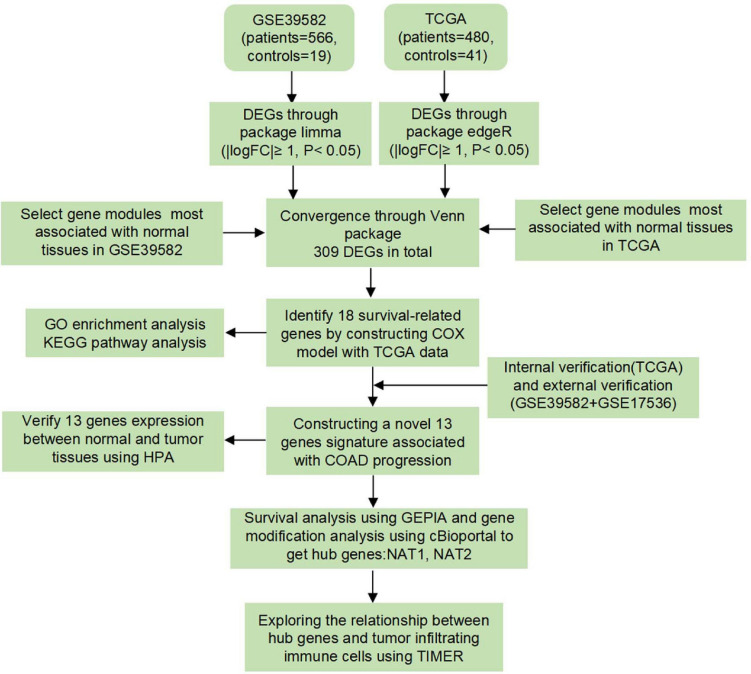
Study design and workflow of this study.

A workflow of this study was depicted in Figure 1. Three independent human COAD datasets obtained from publicly available genomic datasets were included in this study: two expression microarray datasets (GSE39582 and GSE17536) and an RNA-sequencing dataset (TCGA-COAD). From the TCGA-COAD dataset^1^, gene mRNA expression data and the corresponding clinical information from 480 tumor tissues and 41 paracancerous tissues were downloaded, in which the acquisition and application procedures aligned to the protocol. The mRNA-seq data were produced using the Illumina HiSeq 2000 platform and converted to the gene symbols based on the human reference genome hg38. For the expression microarray datasets, original Series Matrix Files of GSE39582 and GSE17536 were collected from the GEO database^2^. GSE39582 was submitted by Marisa et al. (2013) and contained 566 COAD tissues and 19 paracancerous tissues. GSE17536 was submitted by Smith et al. (2010) and consisted of 177 tumor tissues. Owing to the lack of normal tissues, GSE17536 dataset was not included in the next DEG analysis. Detailed information on these datasets is provided in Supplementary Tables 1–3. Standardized data were mapped to the corresponding genetic symbols based on the annotation file provided by the GPL570 platform (Affymetrix Human Genome U133 Plus 2.0 Array). The batch effect of the two-chip data was removed by using an SVA algorithm. Based on the requirement for data integration, data were processed according to the following criteria: (1) data from patients with incomplete information on clinicopathological variables, including survival status and survival time, were removed, and (2) duplicated samples were removed by the average expression values of all these genes.

### Identification of Key Co-expression Modules Using WGCNA

Gene co-expression network analysis was specifically performed on the gene expression profiles of TCGA-COAD and GSE39582 using the “WGCNA” package. The analysis was conducted according to a previous study (Langfelder and Horvath, 2008). First, co-expression analysis was performed for all pair-wise genes using Pearson’s correlation matrices. Subsequently, the weighed adjacency matrix that described the correlation strength between each pair of nodes was constructed by using a power function a_mn_ = |c_mn_| ^β^ (a_mn_ encoded the strength of the correlation between gene m and gene n; c_mn_ represented Pearson’s correlation coefficient between gene m and gene n; β represented a soft-thresholding parameter). After selecting the optimal soft-thresholding power based on the pickSoftThreshold function in R language, the adjacency matrix was transformed into a topological overlap matrix (TOM), which could quantitatively describe the similarity in genes by comparing the weighted correlation between two genes and other genes. Next, hierarchical clustering was conducted to classify genes with similar expression profiles into different gene co-expression modules using the DynamicTreeCut algorithm based on TOM dissimilarity.

To identify candidate modules relevant to clinical traits, module eigengenes (MEs) were obtained using the moduleEigengenes function to indicate the principal component of each module, and the module-trait associations between MEs and clinical subtypes (normal and tumor) were calculated using linear regression. Modules with the highest correlation coefficient among all the selected modules were considered the key modules significantly associated with clinical subtypes of COAD and were subjected to further analysis.

### Identification of DEGs

Screening of DEGs can identify the differences in gene expression levels between tumor tissues and matched normal tissues and identify the specific genes correlated with biological characteristics in tumors. We employed the “edgeR” package to analyze the differences between non-malignant samples and COAD tissues in the TCGA-COAD dataset. The analysis of DEGs in the GSE39582 dataset was conducted using the “limma” package in R software. DEGs including significantly downregulated and upregulated genes were selected for further study with the cut-off criteria of false discovery rate (FDR) < 0.05 and |log_2_ fold change (FC)| > 1 and visualized as volcano plots by using the “ggplot2” package. Afterward, the DEGs were intersected with the co-expression module genes that were extracted from the above mentioned analysis to obtain the overlapping candidate genes (OCGs). Finally, the OCGs were visualized as a Venn diagram using the “VennDiagram” package and subsequently applied to construct a predictive gene signature.

### Construction of Prognostic Signature

The TCGA-COAD dataset served as a training cohort to establish a gene-based model for prognosis prediction of COAD. To determine the feasibility and reliability of survival-associated genes as prognostic markers in COAD, univariate Cox proportional hazards regression analysis was performed to evaluate the associations between the expression of OCGs and patient OS by using the “survival” package. Only those OCGs of the training set with P-values less than 0.05 were selected for stepwise multivariate Cox regression to build a prognostic predictive model. To elucidate the underlying biological mechanisms of survival-associated genes, pathway enrichment analysis including gene ontology (GO) terms and Kyoto Encyclopedia of Genes and Genomes (KEGG) pathways was performed using the “clusterProfiler” package and “org.Hs.eg.db” package. GO terms that consist of the three major classifications—biological process (BP), cellular component (CC), and molecular function (MF)—are able to provide a comprehensive understanding of the biological properties of gene sets for all organisms. The results of GO and KEGG pathway analyses were considered to indicate significance at a cut-off threshold of *P*-value < 0.05, and the “ggplot2” package was applied to visualize the enrichment results to help interpret the results.

Next, the risk score formula of each patient was constructed based on a linear combination of a regression coefficient (β) multiplied by the genetic expression level of significant OCG: The risk score = (β_gene__1_^∗^ expression level of gene1) + (β_gene__2_^∗^ expression level of gene2) + (β_gene__3_^∗^ expression level of gene3) + (β_genen_^∗^ expression level of genen). In addition, univariate and multivariate analyses were performed to determine whether the prognostic value of the prognostic risk model was independent of other clinicopathological parameters including age, gender, stage, and TNM status in the TCGA-COAD dataset.

### Evaluation of the Predictive Value of the Prognostic Signature

To validate the robustness and transferability of the prognostic risk model, the predictive power was validated on the testing cohort. To increase the sample sizes, we merged the GSE39582 and GSE17536 datasets as the testing cohort. With the median risk score as the cut-off value, patients were divided into high-risk and low-risk cohorts according to the gene-based risk score formula. Kaplan–Meier (KM) curves and log-rank tests were plotted to compare two groups’ survival events. The ability of the signature to predict patient survival was further assessed by using receiver operating characteristic (ROC) curve methodology and calculating the area under the curve (AUC) with the R package “survival ROC.” Otherwise, the prognostic risk model was visualized as a risk plot in the training and testing cohorts that comprised the distributions of the risk score, the survival status of each patient and the expression profiles of the screened OCGs.

### Validation of Gene and Protein Expression of Prognostic Genes

Based on the data from the TCGA database, the gene expression levels of prognosis-related genes between COAD and normal tissues were normalized using the “edgeR” package and drawn as a box plot graph. The relationships among prognosis-related genes were analyzed using Pearson correlation analysis and plotted as co-expressed heatmaps in the COAD and normal tissues, respectively. Moreover, the Human Protein Atlas (HPA^3^) was utilized to validate the protein expression levels of prognosis-related genes by immunohistochemistry (IHC).

### Genomic Alterations of Favorable Prognostic Genes by the cBioPortal Database

The cBioPortal Cancer Genomics Portal^4^ is a web-based platform for performing multidimensional cancer genomics data exploration, analytics, and visualization (Gao et al., 2013). The gene alteration status of favorable prognostic genes derived from the prognostic risk model was analyzed using the cBioPortal tool regarding COAD. OncoPrint was constructed in cBioPortal (TCGA provisional) to directly provide an overview of genetic alterations in each gene.

### Survival Analysis of Favorable Prognostic Genes Based on the GEPIA Database

The Gene Expression Profiling Interactive Analysis (GEPIA) database^5^ is a web-based tool for analyzing RNA sequencing expression data and providing customizable functions such as patient survival analysis, which includes 9736 tumors and 8587 normal samples from the TCGA and Genotype-Tissue Expression databases (Tang et al., 2017). Survival curves were plotted using the online tool GEPIA to evaluate the relationship between OS and the expression of favorable prognostic genes in COAD patients.

### Immune Infiltrate Analysis Based on the TIMER Database

TIMER^6^ is a web-based data-mining platform that includes 10,897 samples across 32 cancer types and applies a deconvolution previously published statistical method to determine the relative levels of six immune infiltrates from their gene expression profiles (Li et al., 2017). The association of immune infiltration levels in COAD with somatic copy number alterations (SCNA) for prognostic genes was investigated by the “SCNA module” in the TIMER database. SCNAs in TIMER include deep deletions, arm-level deletions, diploid/normal alterations, arm-level gains and high amplifications. The distributions of each immune cell subset at each copy number status in COAD were plotted by box plots and a two-sided Wilcoxon rank sum test was utilized to compare the immune infiltration level in each SCNA category with that for normal samples. In addition, we further analyzed the correlation of *NAT1* and *NAT2* expression with tumor purity and levels of infiltrating CD8+ T cells and activated myeloid dendritic cells.

### Statistical Analysis

R software (version 3.6.1) was employed to implement the statistical analyses in the study. *P*-values < 0.05 were considered to be significant unless otherwise specified.

## Results

### Construction of Weighted Co-expression Network and Identification of Key Modules

**FIGURE 2:**
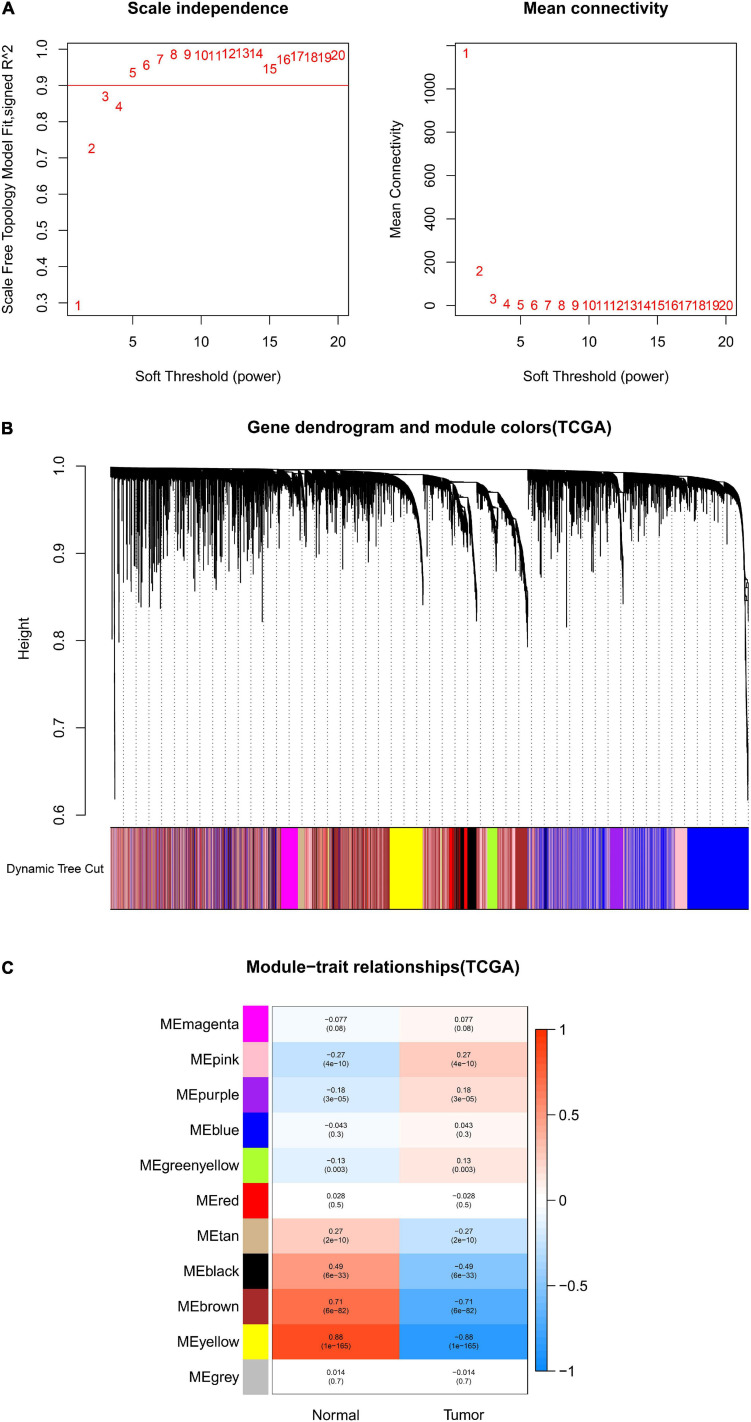
Identification of modules associated with clinical information in the TCGA-COAD dataset. **(A)** Determination of soft-thresholding power in WGCNA analysis. **(B)** Gene cluster tree. Based on the adjacency-based dissimilarity of the hierarchical clustering gene clustering chart, dynamic tree cutting method was utilized to identify modules by dividing the tree diagram at significant branch points. Modules are assigned different colors in the horizontal bar immediately below the tree diagram. **(C)** Module-trait relationships for normal and tumor. Each row in the table corresponds to a color module, and each column to a clinical trait. Numbers in each cell reported the correlation coefficient between each module and clinical traits and the corresponding *p*-value.

**FIGURE 3:**
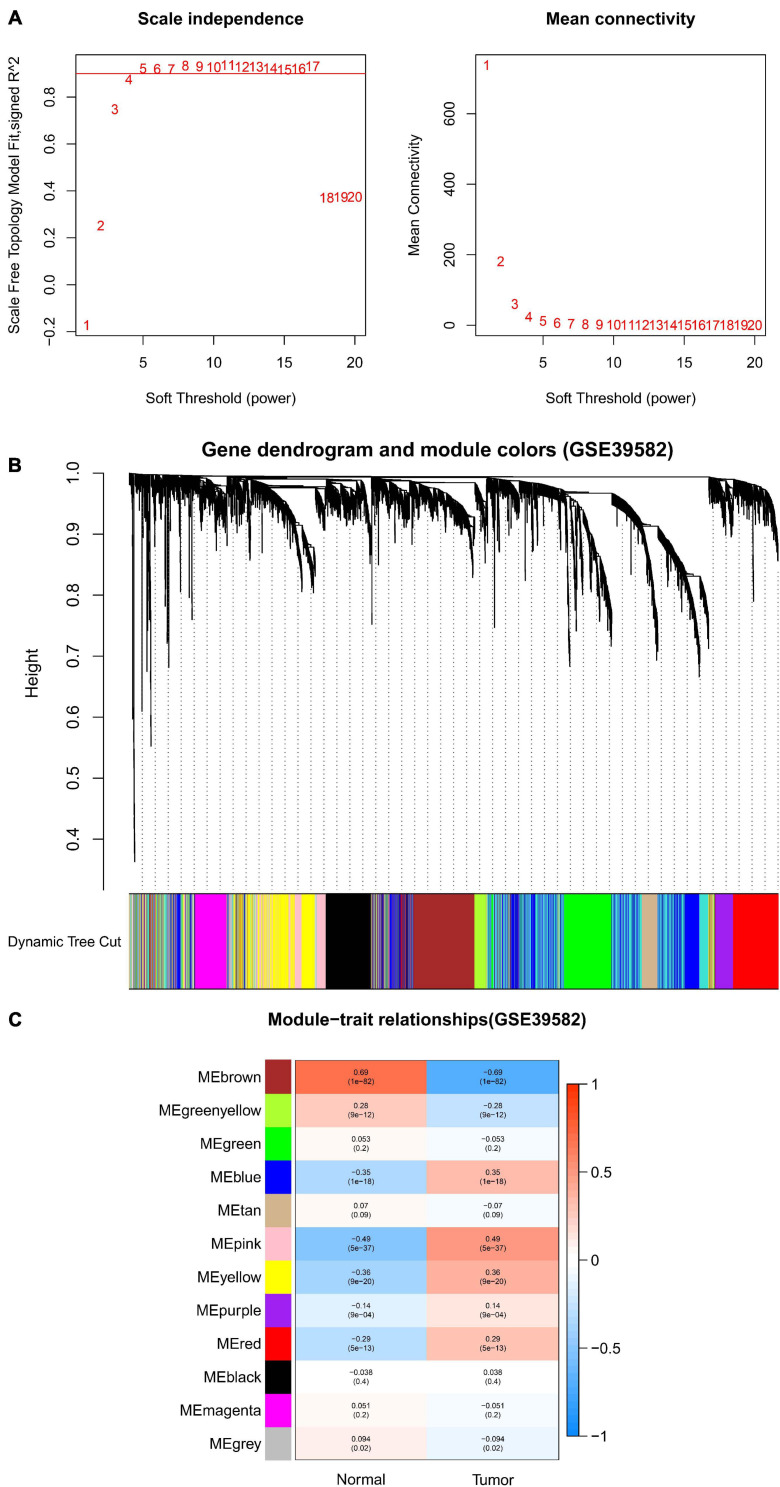
Identification of modules associated with clinical information in the GSE39582 dataset. **(A)** Determination of soft-thresholding power in WGCNA analysis. **(B)** Gene cluster tree. Based on the adjacency-based dissimilarity of the hierarchical clustering gene clustering chart, dynamic tree cutting method was utilized to identify modules by dividing the tree diagram at significant branch points. Modules are assigned different colors in the horizontal bar immediately below the tree diagram. **(C)** Module-trait relationships for normal and tumor. Each row in the table corresponds to a module eigengene, and each column to a clinical characteristic. Numbers in each cell reported the correlation coefficient between each module and clinical traits and the corresponding *p*-value.

After data preprocessing and quality assessment, we obtained the expression matrices from the 521 samples in the TCGA-COAD dataset and the 585 samples in the GSE39582 dataset. Using the system biology method of WGCNA, co-expression modules in COAD patients were identified by constructing the co-expression networks from the TCGA-COAD and GSE39582 datasets. In the present study, a soft power β = 5 (Figure 2A) was chosen to build a scale-free network and 11 modules were generated through average linkage hierarchical clustering in the TCGA-COAD dataset (Figure 2B). Meanwhile, a total of 12 modules (Figure 3B) were obtained by selecting an appropriate soft-thresholding power = 5 in the GSE39582 dataset (Figure 3A). Furthermore, we analyzed the association of modules between each module and clinical subtypes (normal and tumor) to identify key modules and construct the heatmaps of module-trait relationships in Figures 2C, 3C. MEyellow in the TCGA-COAD module (*r* = 0.88, *p* < 0.001) and MEbrown (*r* = 0.69, *p* < 0.001) in the GES39582 module that were found to have the highest association with normal tissues were selected as clinically significant modules.

### Identification of DEGs and OCGs

**FIGURE 4:**
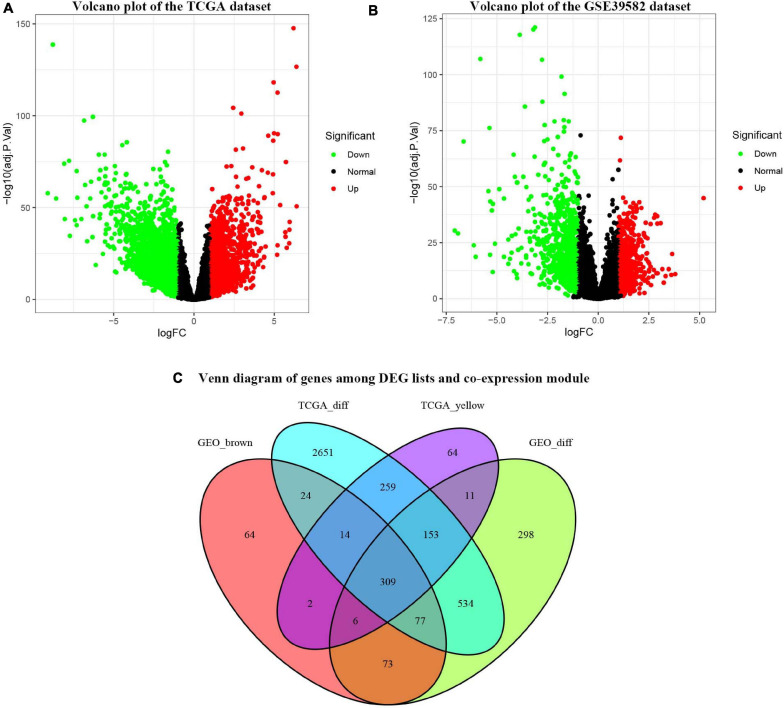
Identification of consensus differentially expressed genes (DEGs) among the TCGA-COAD and GSE39582 datasets of COAD patients. **(A)** Volcano plot of DEGs in the TCGA-COAD dataset. **(B)** Volcano plot of DEGs in the GSE39582 dataset. **(C)** The Venn diagram showing the overlapping candidate genes among DEG lists and co-expression modules.

Under the cut-off criteria of FDR < 0.05 and | logFC| ≥ 1.0, the “limma” algorithm identified 1461 DEGs in the GES39582 dataset (796 upregulated and 665 downregulated genes, Figure 4B). A total of 4021 DEGs in the TCGA-COAD dataset (1609 upregulated and 2412 downregulated genes, Figure 4A) were obtained by the “edgR” package. As plotted in Figure 4C, the brown module of the GES39582 dataset with 569 co-expression genes and the yellow module of the TCGA-COAD dataset with 818 co-expression genes intersected with the DEGs, and 309 genes were screened as the OCGs for further analyzed.

### Identification of a Gene-Based Signature From the Training Dataset

**FIGURE 5:**
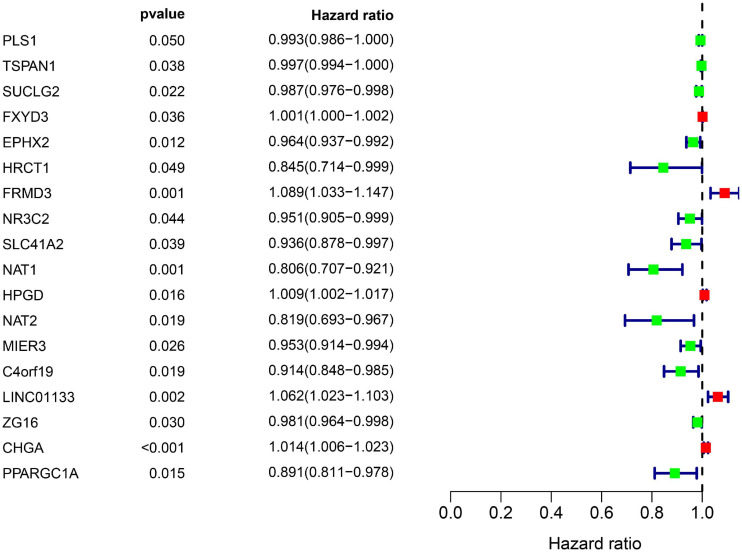
Overall information of 18 prognostic DEGs screened out by univariate Cox proportional hazards regression in the TCGA-COAD dataset. Solid squares represent the hazard ratio (HR) of death, and close-ended horizontal lines represent the 95% confidence intervals (CI).

**FIGURE 6:**
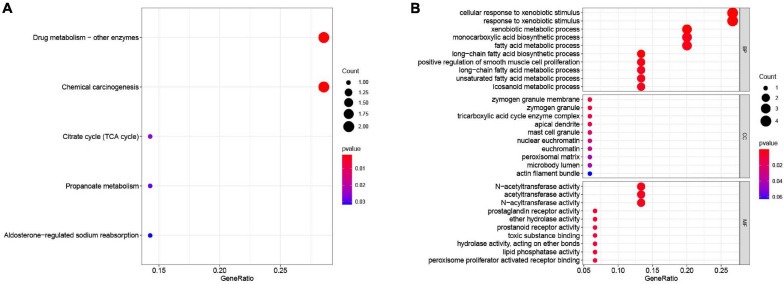
Functional enrichment analysis of the 18 survival-associated genes. **(A)** KEGG pathways in enrichment analysis of the prognostic genes; **(B)** GO enrichment analysis results of the prognostic genes. Bubble color refers to the enrichment *P*-value, and the size of the bubble represents the gene number.

All the OCGs in the training dataset (TCGA-COAD) were subjected to univariate Cox analysis and a total of 18 genes that were significantly associated with OS (Figure 5, *P* < 0.05) were considered to be prognostic genes for multivariate Cox regression analysis. To elucidate the underlying biological mechanisms of 18 survival-related genes, GO and KEGG pathway enrichment analyses were performed using the ClusterProfiler package, and the results demonstrated that 5 KEGG pathways and 241 GO terms were enriched for these prognostic genes (Supplementary Tables 4, 5). The top ten terms in the three functional groups (BP, CC, and MF) from the GO results are demonstrated in Figure 6B. Among the BPs, the prognostic genes were largely associated with metabolic biological processes, including xenobiotic, fatty acid, and icosanoid metabolic processes. For the CC results, it was demonstrated that the prognostic genes were primarily located at zymogen granules, euchromatin, tricarboxylic acid cycle (TCA) enzyme complexes and peroxisomal matrices. Moreover, MF analysis indicated that these genes were primarily involved in regulating the biological functions of multiple enzymes and receptors, such as *N*-acetyltransferase, prostaglandin receptor, hydrolase and peroxisome proliferator activated receptor. According to KEGG analysis (Figure 6A), these genes were correlated with drug metabolism-other enzymes, chemical carcinogenesis and the TCA cycle, which modulated the metabolic biological processes to affect the tumorigenesis of COAD.

Next, 13 genes were further selected to establish a prognostic gene signature, of which four genes were independent prognostic factors associated with unfavorable overall survival (*FXYD3*, *FRMD3*, *LINC01133*, and *CHGA*), and nine genes were confirmed to be favorable prognostic factors for COAD (*TSPAN1*, *HRCT1*, *MIER3*, *NR3C2*, *SLC41A2*, *NAT1*, *NAT2*, *ZG16*, and *PPARGC1A*). The risk score formula for assessing the prognosis of each patient was calculated as follows: risk score = (–0.003) × (expression value of *TSPAN1*) + 0.002 × (expression value of *FXYD3*) + (–0.107) × (expression value of *HRCT1*) + 0.136 × (expression value of *FRMD3*) + (–0.039) × (expression value of *NR3C2*) + (–0.072) × (expression value of *SLC41A2*) + (–0.173) × (expression value of *NAT1*) + (–0.116) × (expression value of *NAT2*) + (–0.033) × (expression value of *MIER3*) + 0.076 × (expression value of *LINC01133*) + (–0.021) × (expression value of *ZG16*) + 0.016 × (expression value of *CHGA*) + (–0.074) × (expression value of *PPARGC1A*). Detailed information on the multivariate Cox regression is presented in Table 1.

**TABLE 1:** Coefficients of 13 genes constituting gene-based risk signature that were identified from multivariate Cox regression analysis.

Gene	Coefficient	HR	HR.95L	HR.95H	*P*-value
*TSPAN1*	–0.003	0.997	0.993	1.001	0.140
*FXYD3*	0.002	1.002	1.001	1.002	0.001
*HRCT1*	–0.107	0.899	0.768	1.054	0.189
*FRMD3*	0.136	1.146	1.070	1.227	0.001
*NR3C2*	–0.039	0.962	0.915	1.011	0.128
*SLC41A2*	–0.072	0.931	0.864	1.002	0.06
*NAT1*	–0.173	0.841	0.734	0.965	0.014
*NAT2*	–0.116	0.890	0.751	1.056	0.181
*MIER3*	–0.033	0.968	0.927	1.011	0.138
*LINC01133*	0.076	1.079	1.042	1.117	<0.001
*ZG16*	–0.021	0.979	0.960	0.998	0.032
*CHGA*	0.016	1.016	1.008	1.023	<0.001
*PPARGC1A*	–0.074	0.929	0.851	1.014	0.099

*HR, Hazard ratio.*

### Prognostic Role of the 13-Gene Signature

**FIGURE 7:**
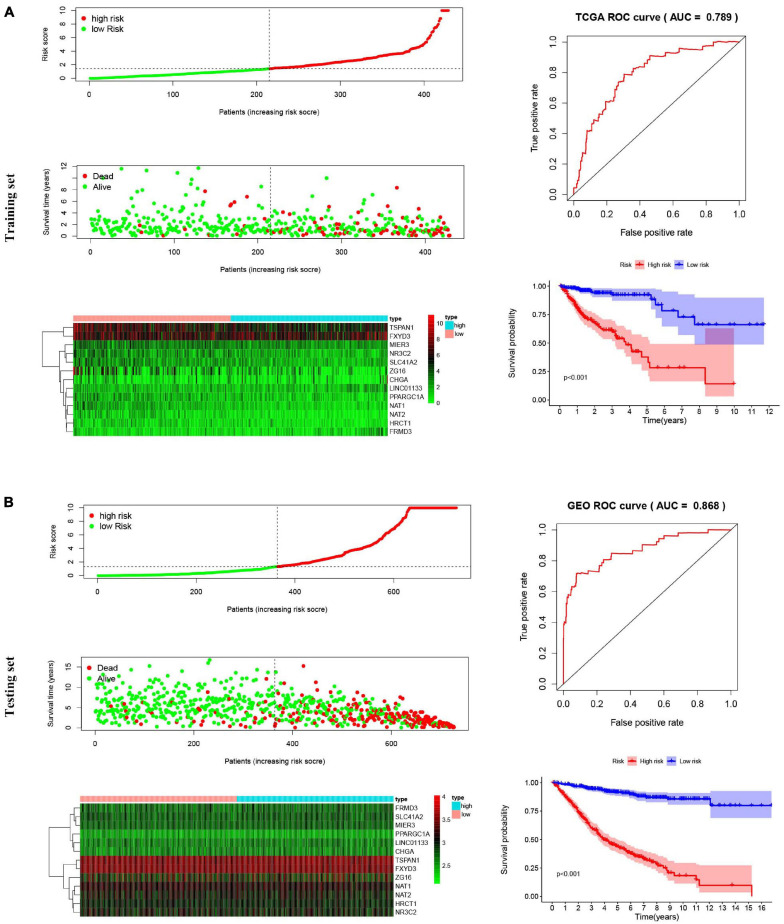
Risk score analysis, Kaplan–Meier survival curves and ROC curves for the 13-gene signature in COAD. Our study employed TCGA-COAD cohort as the training dataset and an independent cohort by merging the GES39582 and GSE17536 datasets as the testing dataset. **(A)** The distributions of the risk score, survival status and expression profiles, Kaplan–Meier curve and ROC curve of the 13-gene signature in the training set. **(B)** The distributions of the risk score, survival status and expression profiles, Kaplan–Meier curve and ROC curve of the 13-gene signature in the testing set.

The 13-gene based risk score was calculated for each patient in the training and testing sets, and patients were stratified into the low-risk and the high-risk subgroups with the median prognostic score of the training set serving as the cut-off point. Next, we used the KM plot and ROC curve to describe the performance of the 13-gene signature in predicting the survival risk of COAD patients. The distribution of the risk score along with the survival status of COAD patients and the heatmap of the 13 prognostic genes in the two datasets are displayed in Figure 7 (left panel), which indicates that patients with low scores had lower mortality rates than did patients with high scores. Consistent with these results, the KM analyses showed that the high-risk group had a significantly shorter OS time than the low-risk group (log-rank *p* < 0.001 in the training and testing sets, Figure 7, right panel). The AUCs for the 13-gene signature reached 0.789 and 0.868 in the training set and the testing set, respectively, indicating the enhanced power of the signature in predicting the survival outcomes of COAD patients (Figure 7, right panel). In addition, we included age as a continuous variable and gender and TNM stage as categorical variables for univariate and multivariable Cox regression analyses to further analyze the performance of our signature in the training set. The results of the multivariate Cox regression analyses showed that the 13-gene signature was an independent and unfavorable prognostic factor in terms of OS after adjusting for age, gender, and TNM stage (HR = 1.015, 95%CI = 1.008–1.022, *p* < 0.001, Table 2).

**TABLE 2:** Identifying the independent prognostic parameters in the TCGA-COAD dataset.

Variables	Univariable model			Multivariable model		
	HR	95%CI of HR	*P*-value	HR	95%CI of HR	*P*-value
13-gene risk score	1.016	1.009-1.023	<0.001	1.015	1.008-1.022	<0.001
Age	1.016	0.995-1.037	0.145	1.035	1.013-1.059	0.002
Gender	1.132	0.704-1.820	0.609	0.968	0.595-1.573	0.895
AJCC stage	3.883	2.309-6.530	<0.001	1.993	0.595-1.573	0.047
ATCC T stage	7.330	1.791-29.996	0.006	3.163	0.743-13.475	0.119
AJCC N stage	4.512	2.790-7.294	<0.001	2.788	1.590-4.888	<0.001
AJCC M stage	3.721	2.300-6.019	<0.001	1.598	0.903-2.829	0.108

*HR, Hazard ratio; CI, confidence interval; AJCC, American Joint Committee on Cancer.*

### Verification of the Expression Patterns of the Prognostic Genes

**FIGURE 8:**
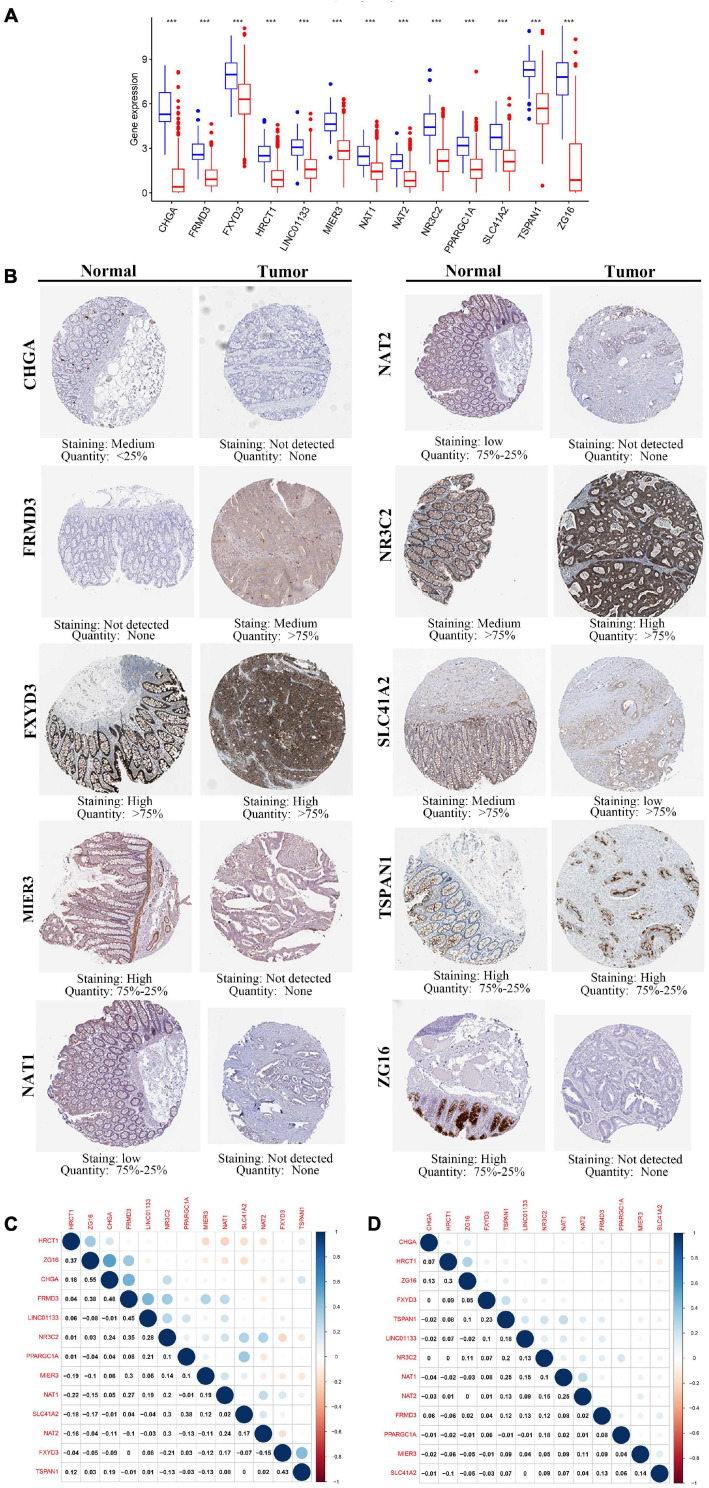
The expression of the 13 prognostic genes in COAD. **(A)** The expression profiles of the 13 genes in the TCGA-COAD dataset. Wilcoxon rank-sum tests were conducted to compare the difference in the expression level of each gene between tumor and normal tissues. ***p < 0.001; N, normal tissues; T, tumor tissues. **(B)** Protein levels of the 13 genes in the COAD and normal tissues based on the Human Protein Atlas. **(C)** Transcription-level correlation analysis of the 13 prognostic genes in the normal tissues of TCGA-COAD dataset. **(D)** Transcription-level correlation analysis of the 13 prognostic genes in the tumor tissues of TCGA-COAD dataset. Pearson correlation analysis was performed to analyze the relationships among prognosis-related genes. Numbers in each cell reported the correlation coefficient between these genes.

To elucidate the role played by the prognostic genes derived from the predictive signature in COAD, we explored the gene expression levels of these genes among the patients of the TCGA database and verified the protein expression levels using the HPA database. As shown in the Figure 8A, all the gene expression levels of prognostic genes were significantly downregulated in COAD compared with non-tumor tissues (All *P*-values < 0.001). The characteristic IHC photos of prognostic genes in tumor and normal tissues are presented in Figure 8B and the results indicated that six of the prognostic genes showed significant downregulation in COAD compared with normal tissue, including *MIER3*, *CHGA*, *SLC41A2*, *NAT1*, *NAT2*, and *ZG16*. However, the HPA dataset did not provide the immunochemical profiles of *HRCT1*, *LINCO1133*, and *PPARGC1A*. Moreover, we employed Pearson correlation analysis to explore the correlation between the mRNA expressions of the 13 prognostic genes in the TCGA dataset. The co-expression pattern in the normal tissues (Figure 8C) was notably different from that in the tumor tissues (Figure 8D).

### Somatic Mutation Landscape and Prognostic Values of Favorable Prognostic Genes

**FIGURE 9:**
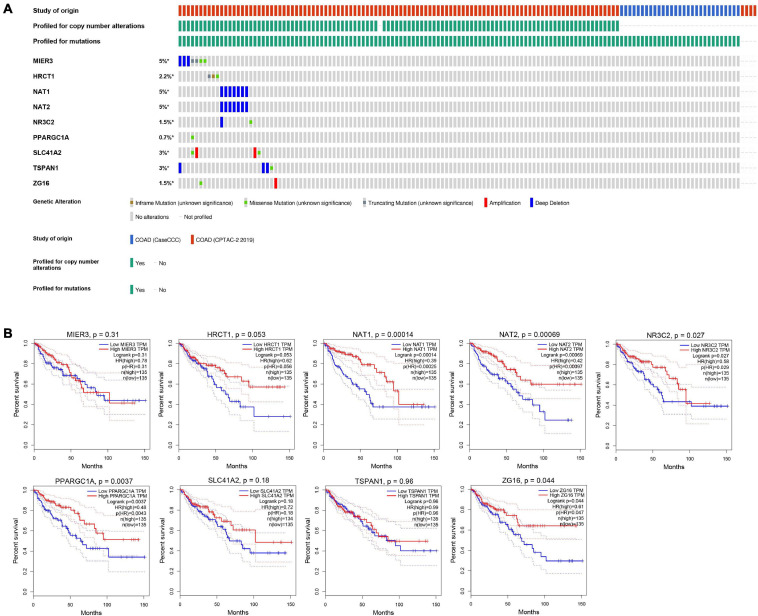
The genomic mutations and prognostic values of the nine favorable prognostic genes in COAD. **(A)** The expression alteration profiles of the nine favorable prognostic genes based on the cBioPortal database. The genetic alterations of the favorable prognostic genes in COAD, including copy number amplification, deep deletion, amplification, and genomic mutation were assessed. The OncoPrint tab provides an overview of genetic alterations in each gene across a sample set. Each row refers to a gene, and each column refers to a tumor sample. **(B)** The overall survival analyses of the 9 favorable prognostic genes using the GEPIA online platform. Kaplan–Meier plotter was applied to evaluate the prognostic value of each gene. Hazard ratios (HRs) and log-rank p-values were calculated.

**FIGURE 10:**
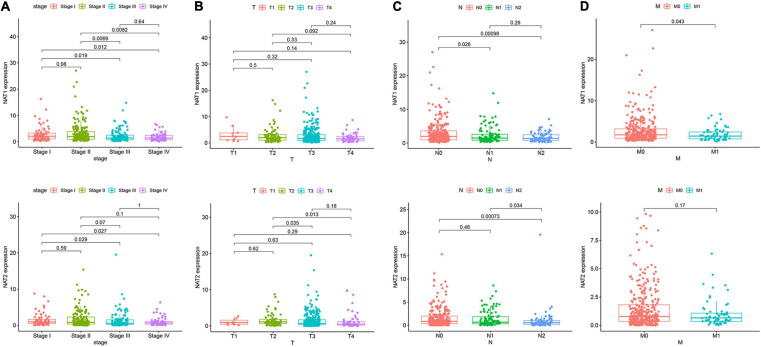
*NAT1* and *NAT2* expression in subgroups of patients with COAD, stratified based on AJCC TNM stage. Wilcoxon rank sum tests were performed to compare the difference of NAT1 and NAT2 expression among different subgroups of clinicopathological variables. **(A)** Boxplot showing relative expression of *NAT1* and *NAT2* in COAD patients in stage 1, 2, 3, or 4. **(B)** Boxplot showing relative expression of *NAT1* and *NAT2* in COAD patients in T1, 2, 3, or 4. **(C)** Boxplot showing relative expression of *NAT1* and *NAT2* in COAD patients in N0, 1, or 2. **(D)** Boxplot showing relative expression of *NAT1* and *NAT2* in COAD patients in M0, or 1. The central mark is the median; the edges of the box are the 25th and 75th percentiles.

Nine genes showing negative coefficients in the prognostic signature were considered to be favorable prognostic genes. Since the tumor genome pattern is reportedly associated with tumorigenesis, we explored the somatic mutation for favorable prognostic genes contained in the prognostic signature by cBioPortal database analysis. Figure 9A illustrates the somatic mutation landscape of the nine favorable prognostic genes in COAD samples, with red and blue representing amplification and deep deletion, respectively. Gene alterations in *MIER3*, *NAT1*, and *NAT2* were observed to occur in 5% of the sequenced cases, and deep deletion accounted for the majority of alteration types. Approximately 3% of genetic alterations of *TSPAN1* were observed in COAD patients, including deep deletions and missense mutations with unknown significance. Moreover, copy number alterations (CNAs) were found in the most of COAD patients. In addition, OS analyses of the nine favorable prognostic genes were conducted by KM analyses based on the GEPIA database to further confirm the prognostic values of these genes in patients with COAD (Figure 9B). Among these genes, *NAT1*, *NAT2*, *NR3C2*, *ZG16*, and *PPARGC1A* showed significant positive correlations with OS and could be considered to be protective genes in COAD. From the above mentioned analyses, we found that only the two protective genes *NAT1* and *NAT2* underwent the deep deletion and tended to be downregulated in COAD tissues, suggesting that the two genes might play critical roles in cancer development and progression. Furthermore, we compared the differences in *NAT1* and *NAT2* among different subgroups in COAD (Figure 10). *NAT1* and *NAT2* were significantly differentially expressed in COAD patients with different AJCC stages. Lower *NAT1* and *NAT2* expression was associated with higher pathological stage.

### Association of *NAT1* and *NAT2* Expression With Immune Infiltration

**FIGURE 11:**
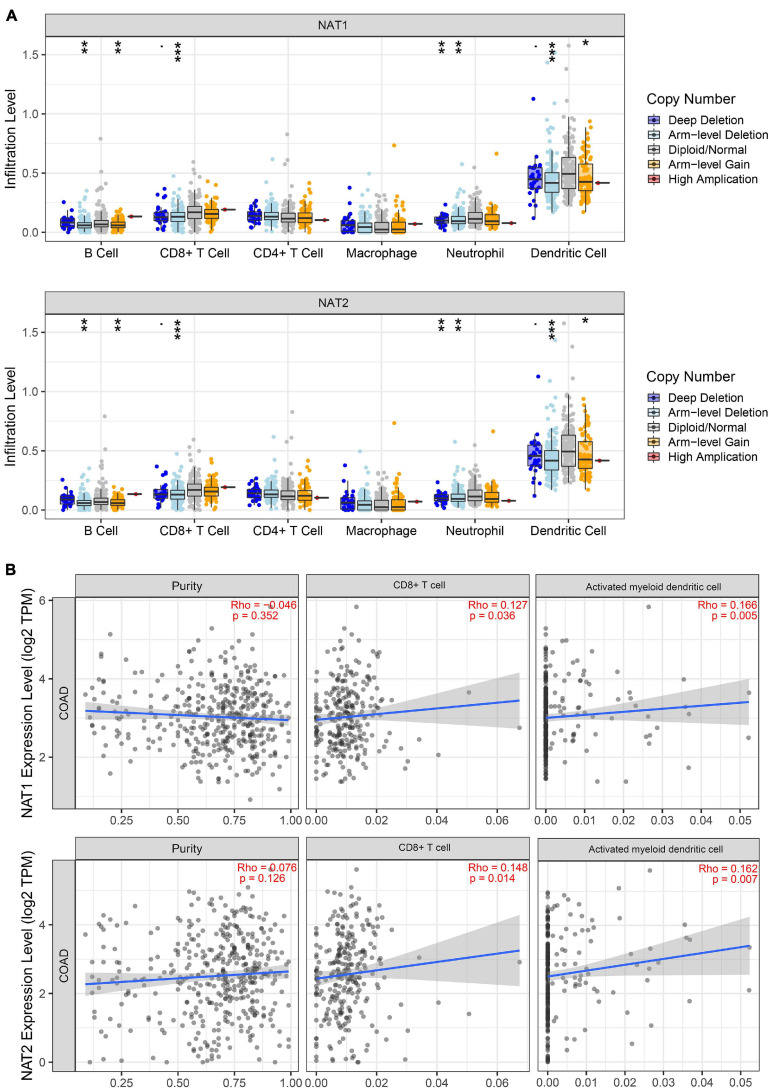
TIMER analyses of *NAT1* and *NAT2*. **(A)** The associations of somatic copy number alterations (SCNAs) of *NAT1* and *NAT2* with immune infiltrates in COAD. The SCNAs in TIMER included deep deletions, arm-level deletions, diploid/normal alterations, arm-level gains and high amplifications. The SCNA categories of *NAT1* and *NAT2* were presented at the right bottom and the distributions of each immune cell subset at each mutation status were plotted by box plots. Two-sided Wilcoxon rank sum test with calculated *p*-value was utilized to compare the immune infiltration level in each category with that for normal samples. *P < 0.05; **P < 0.01; ***P < 0.001. **(B)** Correlation of *NAT1* and *NAT2* expression levels with tumor purity and infiltrating levels of CD8+ T cells and activated myeloid dendritic cells. The Spearman method was used to determine the correlation coefficient. *NAT1* and *NAT2* expression levels were plotted on the *y*-axis, while the abundance of immune infiltrating cells was plotted on the *x*-axis. Gene expression levels against tumor purity was displayed on the left-most panel.

It is well-known that immune cells play an important anti-tumor surveillance role. Thus, to elucidate the potential regulatory mechanisms of *NAT1* and *NAT2* in the development of COAD, the relationships between the SCNAs of *NAT1* and *NAT2* and immune infiltrates in the COAD microenvironment were explored. Compared to the immune infiltrate levels of six cells, deletion of *NAT1* and *NAT2* was associated with substantially lower levels of four immune cell types, including B cells, CD8+ T cells, neutrophils, and dendritic cells, which indicated their influence on the tumor microenvironment (Figure 11A). Furthermore, we observed that *NAT1* and *NAT2* expression was significantly correlated with the infiltration levels of CD8+ T cells and dendritic cells (Figure 11B).

## Discussion

The molecular pathogenesis of COAD is multifaceted in nature and characterized by a variety of genomic instabilities, epigenomic alterations, gene expression dysregulation and chromosomal aberrations, which are not separate events but multiple cellular processes (Cancer Genome Atlas Network, 2012; Guinney et al., 2015). Although several advances focusing on diagnostic and therapeutic techniques have been identified to effectively reduce the mortality rates of COAD patients, there are still a number of challenges facing early diagnostic and therapeutic strategies, including a lack of the awareness of high-risk patients, a lack of clinically applicable biomarkers to identify high-risk patients, and the high cost of screening high-risk populations. Currently, genes can be utilized to construct a prognostic risk model that helps to assess tumor progression, prognosis and reaction to therapeutic strategies, and a number of studies have established gene signatures based on large-scale public datasets (Zuo et al., 2019; Yuan et al., 2020a). Therefore, to accurately predict survival time and identify high-risk patients, we conducted a comprehensive screening of DEGs from two independent datasets and subsequently constructed a 13-gene signature in prognosis prediction for COAD patients. We also performed validation analysis of the prognostic predictive signature and found that this signature was credible in predicting the OS of COAD patients.

Compared with the gene-based signatures constructed in the previous study (Zuo et al., 2019; Yuan et al., 2020a), our prognostic model was different. First, we adopted integrated bioinformatic methods, WGCNA and DEG analysis, to select significant DEGs related to the clinical traits from the GES39582 dataset and the TCGA-COAD dataset. Integrated bioinformatic analysis tends to be an effective method to identify tumor-specific genetic alterations associated with the occurrence and development of tumors and guide patients’ personalized therapy. Although traditional DEGs analysis is a powerful analysis that can discover genetic alterations between control groups and experimental groups, then generating highly valuable information, only WGCNA, a data exploration tool, can be used to determine the interactions among genes and find modules of highly related genes that are significantly associated with clinical features and biological tumor behavior. Second, numerous studies have used WGCNA to select key modules associated with clinicopathological parameters in multiple cancers. For example, Xie and Xie (2019) identified genes significantly associated with pathological M stage based on WGCNA and constructed a 6-gene signature for the prognosis of non-small-cell lung cancer patients. A previous study defined one gene module related to tumor grades in colorectal cancer, and the putative representative biomarkers associated with prognosis were identified (Yuan et al., 2020b). Unlike traditional WGCNA, our study focused on the modules notably correlated with normal tissues in the two independent datasets and selected the module genes that might play an important role in maintaining physiological function. Thus, our study identified a brown module in the GES39582 dataset and a yellow module in the TCGA-COAD dataset, both of which were significantly related to normal tissues compared with tumor tissues. Furthermore, the 309 OCGs between DEGs and the co-expression module genes were obtained and subjected to univariate and multivariate Cox analyses for prognostic signature construction. Our study employed TCGA cohort as the training dataset and an independent cohort by merging the GES39582 and GSE17536 datasets as the testing dataset. Moreover, to minimize variability, an SVA algorithm was utilized to remove the batch effect of the two GEO datasets.

In this study, a total of 18 survival-related genes was firstly identified based on univariate Cox analysis in the TCGA-COAD dataset. Functional annotation analysis indicated that these genes were mainly involved in various metabolic processes, which might affect the development of cancer. The top activated pathway in the enrichment analysis was fatty acid metabolic process, an essential cellular process that reflects the function of mitochondria. Increased fatty acid synthesis is crucial for the proliferation and growth of cancer cells by new membrane biosynthesis and steroid hormone production, thereby promoting tumorigenesis and tumor progression (Röhrig and Schulze, 2016). Next, we constructed a novel gene-based signature consisting of 13 genes (*FXYD3*, *MIER3*, *LINC01133*, *CHGA*, *TSPAN1*, *HRCT1*, *FRMD3*, *NR3C2*, *SLC41A2*, *NAT1*, *NAT2*, *ZG16*, and *PPARGC1A*) for predicting the OS of COAD patients. Furthermore, the 13-gene signature could categorize COAD patients into low-risk and high-risk groups with statistically different survival outcomes, which was validated by the ROC analysis and KM curve analysis in both TCGA and the merged GEO datasets. Besides, to further clarify whether this signature is an independent factor in COAD, multivariate Cox regression analyses was performed and showed that it was able to predict the survival of COAD patients without consideration of other conventional clinicopathological variables, including age, gender, and AJCC stage. Taken together, these findings provide the evidence for translating the 13-gene signature into clinical practice.

In the 13-gene signature, most genes were regarded as favorable prognostic genes, while only *FXYD3*, *FRMD3*, *LINC01133*, and *CHGA* were found to do the opposite. As the survival time of cancer patients could be influenced by aberrant expression of genes, we confirmed the gene and protein expression patterns of the prognostic genes based on the TCGA database and HPA database. All 13 genes were determined to be downregulated at the genetic level in COAD tissues relative to normal samples, among which six genes were consistent with the IHC results in the HPA dataset and tended to be reduced at the protein level in tumor specimens, including *MIER3*, *CHGA*, *SLC41A2*, *NAT1*, *NAT2*, and *ZG16*, providing the vital function of favorable prognostic genes in COAD. However, unfavorable prognosis-related genes have also been reported to be involved in tumor proliferation. *FXYD3*, a new regulator of Na-K-ATPase, has been found to be expressed in normal colon tissues (Geering, 2006). A study on a total of 150 resected colorectal cancer specimens measured the protein levels of *FXYD3* by IHC staining and demonstrated an association of downregulated expression of *FXYD3* proteins with cancer progression defined by Dukes’ staging (Widegren et al., 2009). Recent publications have revealed that *LINC01133* is significantly reduced in colorectal cancer and is considered as a potential tumor suppressor in cancer progression and metastasis (Kong et al., 2016; Zhang et al., 2017). *CHGA* has been approved as a powerful biomarker for the early detection of various digestive system carcinomas, including gastric cancer (Yang and Chung, 2008), pancreatic neuroendocrine tumors (Weisbrod et al., 2013), and colorectal cancer (Zhang et al., 2019). The current research mechanism of *FRMD3* in COAD has not been reported to date, but it has been reported that non-small cell lung carcinoma (NSCLC) is highly correlated with reduced *FRMD3* expression, which could induce apoptosis by regulating the activity of caspases in NSCLC. Therefore, further research is warranted to be carried out to characterize the role of *FRMD3* in COAD.

For the favorable prognostic genes, their genetic status was further analyzed by the cBioPortal tool. The results showed that deep deletion was the most common genetic alteration, which could result in gene expression downregulation in tumors, further indicating the credibility of our results. Various studies have suggested that these favorable prognostic genes might play important roles in tumor progression. A recent study showed that *MIER3* expression was significantly reduced in colorectal cancer at the mRNA and protein levels and was negatively correlated with aggressive tumors and poor clinical outcomes (Peng et al., 2017). Moreover, overexpression of *MIER3* could inhibit the aggressive behaviors of colorectal cancer *in vivo* and *in vitro* (Peng et al., 2017). In our study, the mRNA and protein levels of *MIER3* were significantly reduced in tumor tissues, and deep deletion was the most common type of *MIER3* mutation in COAD. However, no correlation was found between the gene expression of *MIER3* and the prognosis of COAD patients in our survival analysis. *TSPAN1*, a member of the transmembrane 4 superfamily, has been reported to be increased in various cancers at the mRNA level, including prostate cancer (Xu et al., 2000), gastric carcinoma (Chen et al., 2008), and COAD (Chen et al., 2009). A clinical study indicated that COAD patients with *TSPAN1* overexpression had a significantly shorter survival period than patients with weak expression, which was not consistent with our survival study (Chen et al., 2009). An *in vitro* study indicated that the downregulation of *TSPAN1* significantly inhibited the proliferation and invasion of colon cancer cells, suggesting that *TSPAN1* might be a valuable therapeutic target molecule in colon cancer (Chen et al., 2010). Thus, the molecular mechanisms governing *TSPAN1* in COAD still need to be further investigated. Zymogen granule protein 16 (*ZG16*) is primarily expressed in mucus-secreting cells, including goblet cells in the colon (Tateno et al., 2012). In a clinical study with a small sample size, *ZG16* expression was found to be sequentially downregulated from normal colon tissues and neoplastic precursor adenomatous polyps to COAD tissues (Meng et al., 2018). A recent study showed that the expression of *ZG16* was associated with distant metastasis and lymphatic invasion in colorectal cancer (Meng et al., 2020). In concordance with previous studies, our study found that the gene and protein expression levels of *ZG16* were significantly reduced in tumor tissues and correlated with poor prognosis, supporting the tumor suppressor role of *ZG16* in COAD progression. *PPARGC1A* is a transcriptional coactivator of the *PGC-1* gene family that modulates the process of energy metabolism and mitochondrial biogenesis (Seale, 2015). Based on the survival analysis in the GEPIA database, we found that patients with higher *PPARGC1A* expression had a better prognosis in COAD. However, the effect of *PPARGC1A* on the initiation and progression of colorectal cancer remains controversial. Accumulating studies have shown that *PPARGC1A* promoted tumor growth (Bhalla et al., 2011; Vellinga et al., 2015), whereas several studies have found that the lower expression of this gene in COAD is associated with an increased risk of cancer (Feilchenfeldt et al., 2004). In another study, genetic polymorphisms in *PPARGC1A* (*rs3774921*) increased the risk of colorectal cancer in individuals fed a highly inflammatory diet (Cho et al., 2017). *NR3C2* is a mineralocorticoid receptor gene encoding mineralocorticoid receptor (MR) that has been considered a tumor suppressor in colorectal cancer, which is consistent with our study (Tiberio et al., 2013). MR downregulation in colorectal cancer was correlated with increased expression of the *VEGF* receptor, indicating that *NR3C2* exerted specific role in decreasing angiogenesis in tumor (Tiberio et al., 2013). *HRCT1* and *SLC41A2* were not reported to be involved in the process of tumorigenesis. Further studies are needed to decipher the biological functions of *HRCT1* and *SLC41A2* in COAD.

*NAT1* and *NAT2* are two members of the *N*-acetyltransferases (*NAT*) family that encode polymorphic enzymes catalyzing the metabolic activation of heterocyclic aromatic amines (HCAs), which have been considered established carcinogens in human colorectal cancer and urinary bladder cancer (Kadlubar et al., 1992; Cross and Sinha, 2004). GO enrichment analysis of the prognostic genes showed that these genes were closely related to *N*-acetyltransferase activity, which was consistent with the biological functions of *NAT1* and *NAT2*. Previous studies have shown that individuals with polymorphisms in *NAT1* or *NAT2* enzymes were susceptible to HCAs present in tobacco smoke and high-temperature cooked meat (Keku et al., 2003; Nöthlings et al., 2009). For example, *NAT1* and *NAT2* acetylator status might create predispositions to increased COAD risk with exposure to tobacco smoke and meat consumption (Lilla et al., 2006). Although most studies have focused on the role of *NAT1* and *NAT2* genetic polymorphisms in COAD risk, the potential role played by their aberrant expression in COAD has largely been ignored and whether *NAT1 and NAT2* expression influences cancer patient survival remains unknown. Liu et al. identified *NAT1* and *NAT2* as critical downregulated genes for CRC, but this study was limited by a small sample size (Liu et al., 2015). Consistent with the previous study, we found that the expressions of *NAT1* and *NAT2* was significantly reduced in tumor tissues at the mRNA and protein levels, possibly attributable to the highly frequent deep deletion of both genes in COAD, which was confirmed by cBioPortal analysis. Moreover, we used the online tool GEPIA to analyze the prognostic values of *NAT1 and NAT2* expression and found that lower levels of *NAT1 and NAT2* expression were correlated with poorer prognosis in COAD patients. These findings suggested that *NAT1 and NAT2* might play novel tumor suppressor roles in the development and metastasis of COAD and could be served as prognostic biomarkers in COAD.

Previous studies have shown that *NAT1* is expressed predominantly on T cells while *NAT2* is expressed in macrophages and natural killer cells, responsible for the adaptive and innate immune response (Salazar-González et al., 2014, 2018). The possible roles played by *NAT1* and *NAT2* in modulating the immune response in COAD have not been determined to date. Hence, we explored the relationship between *NAT1* and *NAT2* expression and the infiltration levels of immune cells and found that deletion of *NAT1* and *NAT2* was associated with substantially lower levels of immune cells, including B cells, CD8+ T cells, neutrophils, and dendritic cells. Moreover, positive relationships between *NAT1* and *NAT2* expression levels and infiltration levels of CD8+ T cells and dendritic cells were identified. It is well-known that neoantigens accumulating on tumor cells are initially recognized and presented by dendritic cells, subsequently promoting the production of CD8+ T cells, which are considered the main executors of cancer destruction, enhancing immune cell activities in the microenvironment, and thus preventing the development of cancer (Chen and Flies, 2013; Buoncervello et al., 2019). These results supported the notion that *NAT1* and *NAT2* downregulation might inhibit the antitumor immune response, enhancing tumor cell invasion and metastasis and thus decreasing the survival time of cancer patients. However, this hypothesis needs to be further validated.

Inevitably, there are several limitations in the present study. First, a major issue is that we did not collect patients diagnosed with COAD with adequate information in our own hospital to validate the predictive performance of the 13-gene based signature. A GEO cohort was used to confirm the robustness of this signature, which could make up for it slightly. Second, all of our samples and clinical data were based on the TCGA and GEO datasets, in which most patients were Western patients. Cohorts with larger sample sizes from other regions are warranted to extend our findings. Third, the prognostic risk model comprised too many genes, which might decrease the accuracy of the model and increase the expenses of laboratory testing, thereby limiting its clinical application. Moreover, although we performed a comprehensive bioinformatic analysis to build a prognostic risk model, the results of bioinformatic analysis can be biased to an extent when analyzing the data that have fewer non-tumor tissues than tumor tissues or addressing technical artifacts of WGCNA, which is similar to the limitations of other bioinformatic methods. Thus, large sample sizes of normal tissues will be important for reliable interpretation of data. In consideration of the credibility of the WGCNA results, TCGA data and IHC data from the HPA database were employed to confirm the gene and protein expression levels of the prognostic genes. However, due to the limitations of the HPA dataset, the IHC results of some prognostic genes in COAD patients were lacking. A series of experiments should be performed to clarify the underlying mechanism of the prognostic genes in the regulation of tumorigenesis in COAD.

In this study, we identified a 13-gene prognostic signature to predict the OS of COAD by using a series of bioinformatics analyses, which could accurately separate COAD patients with unfavorable prognoses from those with favorable prognoses. Moreover, the prognostic genes derived from the predictive signature have the potential to modulate the tumorigenesis and progression of COAD, especially *NAT1* and *NAT2*, which have been implicated in modulating antitumor immunity. Therefore, the results of the present study not only showed the value of the 13-gene signature as a promising classification tool for COAD prognosis but also provided new insights into the role of *NAT1* and *NAT2* in the tumorigenesis and progression of COAD.

## Data Availability Statement

Publicly available datasets were analyzed in this study. This data can be found here: TCGA repository (http://cancergenome.nih.gov/) and GEO (https://www.ncbi.nlm.nih.gov/geo/).

## Ethics Statement

The studies involving human participants were reviewed and approved by China-Japan Friendship Hospital (No. 2018-116-K85-1). Written informed consent for participation was not required for this study in accordance with the national legislation and the institutional requirements.

## Author Contributions

CZ designed the study and performed the data analysis. ZZ and HL took part in analyzing the data. SY revised the manuscript. DZ designed the study and wrote the manuscript. All authors contributed to the article and approved the submitted version.

## Conflict of Interest

The authors declare that the research was conducted in the absence of any commercial or financial relationships that could be construed as a potential conflict of interest.

## Footnotes

1 https://portal.gdc.cancer.gov/repository

2 https://www.ncbi.nlm.nih.gov/geo/

3 http://www.proteinatlas.org

4 http://cbioportal.org

5 http://gepia.cancer-pku.cn/

6 https://cistrome.shinyapps.io/timer/
