# Supplementary material for: Weighted Gene Co-expression Network Analysis Identified a Novel Thirteen-Gene Signature Associated With Progression, Prognosis, and Immune Microenvironment of Colon Adenocarcinoma Patients
Source: Front Genet. 2021 Jul 12;12:657658. doi: 10.3389/fgene.2021.657658 (PMC8312261; doi:10.3389/fgene.2021.657658)
Supplement: Supplementary file 1 [file Table_1.pdf]

Supplementary Table 1 The clinical information of COAD patients in TCGA-COAD dataset.

**Number of patients**	521
**Median Age (years)**	68
**Histology Type**	
**Tumor**	480
**Normal**	41
**Gender**	
**Male**	243
**Female**	216
**Pathological TNM Stage**	
**Stage I**	76
**Stage II**	178
**Stage III-IV**	194
**NA**	11
**Pathological Tumor (T)**	
**T1**	11
**T2**	78
**T3**	313
**T4**	57
**Pathological Lymph Node (N)**	
**N0**	270
**N1**	106
**N2**	83
**Pathological Metastasis (M)**	
**M0**	337
**M1**	65
**NA**	57
**Overall event**	
**Death**	92
**Alive**	367
