# Supplementary material for: Weighted Gene Co-expression Network Analysis Identified a Novel Thirteen-Gene Signature Associated With Progression, Prognosis, and Immune Microenvironment of Colon Adenocarcinoma Patients
Source: Front Genet. 2021 Jul 12;12:657658. doi: 10.3389/fgene.2021.657658 (PMC8312261; doi:10.3389/fgene.2021.657658)
Supplement: Supplementary file 2 [file Table_2.pdf]

Supplementary Table 2 The clinical information of COAD patients in GSE39582 dataset.

Number of patients	585
**Median Age (years)**	69
**Gender**	
**Male**	332
**Female**	263
**Histology Type**	
**Tumor**	566
**Normal**	19
**Pathological TNM Stage**	
**Stage I**	38
**Stage II**	271
**Stage III-IV**	270
**NA**	6
**Pathological Tumor (T)**	
**T1-2**	61
**T3**	379
**T4**	119
**NA**	26
**Pathological Lymph Node (N)**	
**N0**	314
**N1**	137
**N2-3**	106
**NA**	68
**Pathological Metastasis (M)**	
**M0**	499
**M1**	61
**NA**	25
**Overall event**	
**Death**	194
**Alive**	385
**NA**	6
