# Supplementary material for: Weighted Gene Co-expression Network Analysis Identified a Novel Thirteen-Gene Signature Associated With Progression, Prognosis, and Immune Microenvironment of Colon Adenocarcinoma Patients
Source: Front Genet. 2021 Jul 12;12:657658. doi: 10.3389/fgene.2021.657658 (PMC8312261; doi:10.3389/fgene.2021.657658)
Supplement: Supplementary file 3 [file Table_3.pdf]

Supplementary Table 3 The clinical information of COAD patients in GSE17536 dataset.

**Number of patients**	177
**Median Age (years)**	66
**Gender**	
**Male**	96
**Female**	81
**Race**	
**Caucasian**	151
**Black**	9
**Other**	17
**AJCC Stage**	
**Stage I**	24
**Stage II**	57
**Stage III-IV**	96
**Grade**	
**Poorly differentiated**	27
**Moderately differentiated**	134
**Well differentiated**	16
**Recurrence**	
**Recurrence**	35
**No recurrence**	109
**NA**	32
**Overall event**	
**Death**	73
**Alive**	104
