# Supplementary material for: Weighted Gene Co-expression Network Analysis Identified a Novel Thirteen-Gene Signature Associated With Progression, Prognosis, and Immune Microenvironment of Colon Adenocarcinoma Patients
Source: Front Genet. 2021 Jul 12;12:657658. doi: 10.3389/fgene.2021.657658 (PMC8312261; doi:10.3389/fgene.2021.657658)
Supplement: Supplementary file 4 [file Table_4.pdf]

Supplementary table 4 KEGG enrichment analysis of the thirteen prognostic genes derived from the predictive signature in COAD.

ID	Description	Count	p-value	q-value	Gene ID
**hsa00983**	Drug metabolism - other enzymes	2	0.002	0.014	NAT1/NAT2
**hsa05204**	Chemical carcinogenesis	2	0.002	0.014	NAT1/NAT2
**hsa00020**	Citrate cycle (TCA cycle)	1	0.026	0.087	SUCLG2
**hsa00640**	Propanoate metabolism	1	0.029	0.087	SUCLG2
**hsa04960**	Aldosterone-regulated sodium reabsorption	1	0.032	0.087	NR3C2
