# Supplementary material for: Weighted Gene Co-expression Network Analysis Identified a Novel Thirteen-Gene Signature Associated With Progression, Prognosis, and Immune Microenvironment of Colon Adenocarcinoma Patients
Source: Front Genet. 2021 Jul 12;12:657658. doi: 10.3389/fgene.2021.657658 (PMC8312261; doi:10.3389/fgene.2021.657658)
Supplement: Supplementary file 5 [file Table_5.pdf]

Supplementary table 5 GO enrichment analysis of the thirteen prognostic genes derived from the predictive signature in COAD.

ONTOLOGY	ID	Description	Count	p-value	q-value	Gene ID
**BP**	GO:0071466	cellular response to xenobiotic stimulus	4	0.000	0.004	EPHX2/NAT1/NAT2/PPARGC1A
**BP**	GO:0009410	response to xenobiotic stimulus	4	0.000	0.013	EPHX2/NAT1/NAT2/PPARGC1A
**BP**	GO:0006805	xenobiotic metabolic process	3	0.000	0.015	EPHX2/NAT1/NAT2
**BP**	GO:0042759	long-chain fatty acid biosynthetic process	2	0.000	0.024	EPHX2/HPGD
**BP**	GO:0072330	monocarboxylic acid biosynthetic process	3	0.002	0.068	EPHX2/HPGD/PPARGC1A
**BP**	GO:0048661	positive regulation of smooth muscle cell proliferation	2	0.003	0.068	HPGD/PPARGC1A
**BP**	GO:0006631	fatty acid metabolic process	3	0.003	0.068	EPHX2/HPGD/PPARGC1A
**BP**	GO:0001676	long-chain fatty acid metabolic process	2	0.003	0.068	EPHX2/HPGD
**BP**	GO:0033559	unsaturated fatty acid metabolic process	2	0.003	0.068	EPHX2/HPGD
**BP**	GO:0006690	icosanoid metabolic process	2	0.004	0.068	EPHX2/HPGD
**BP**	GO:0019218	regulation of steroid metabolic process	2	0.004	0.068	EPHX2/PPARGC1A
**BP**	GO:0032355	response to estradiol	2	0.005	0.068	HPGD/PPARGC1A
**BP**	GO:0007586	digestion	2	0.005	0.068	PLS1/PPARGC1A

**BP**	GO:0046394	carboxylic acid biosynthetic process	3	0.005	0.068	EPHX2/HPGD/PPARGC1A
**BP**	GO:0016053	organic acid biosynthetic process	3	0.006	0.068	EPHX2/HPGD/PPARGC1A
**BP**	GO:0035296	regulation of tube diameter	2	0.006	0.068	EPHX2/CHGA
**BP**	GO:0050880	regulation of blood vessel size	2	0.006	0.068	EPHX2/CHGA
**BP**	GO:0097746	regulation of blood vessel diameter	2	0.006	0.068	EPHX2/CHGA
**BP**	GO:0035150	regulation of tube size	2	0.006	0.068	EPHX2/CHGA
**BP**	GO:0006633	fatty acid biosynthetic process	2	0.007	0.068	EPHX2/HPGD
**BP**	GO:1901568	fatty acid derivative metabolic process	2	0.008	0.068	EPHX2/HPGD
**BP**	GO:0048660	regulation of smooth muscle cell proliferation	2	0.008	0.068	HPGD/PPARGC1A
**BP**	GO:0035865	cellular response to potassium ion	1	0.008	0.068	PPARGC1A
**BP**	GO:0071313	cellular response to caffeine	1	0.008	0.068	PPARGC1A
**BP**	GO:0048659	smooth muscle cell proliferation	2	0.008	0.068	HPGD/PPARGC1A
**BP**	GO:0003018	vascular process in circulatory system	2	0.008	0.068	EPHX2/CHGA
**BP**	GO:0050821	protein stabilization	2	0.009	0.068	TSPAN1/PPARGC1A
**BP**	GO:00468	positive regulation of hormone biosynthetic process	1	0.009	0.068	PPARGC1A

	86					
**BP**	GO:0071372	cellular response to follicle-stimulating hormone stimulus	1	0.009	0.068	PPARGC1A
**BP**	GO:0086023	adenylate cyclase-activating adrenergic receptor signaling pathway involved in heart process	1	0.009	0.068	CHGA
**BP**	GO:1901077	regulation of relaxation of muscle	1	0.009	0.068	CHGA
**BP**	GO:0008217	regulation of blood pressure	2	0.009	0.068	EPHX2/CHGA
**BP**	GO:0043401	steroid hormone mediated signaling pathway	2	0.009	0.068	NR3C2/PPARGC1A
**BP**	GO:0002551	mast cell chemotaxis	1	0.010	0.068	CHGA
**BP**	GO:0014889	muscle atrophy	1	0.010	0.068	PPARGC1A
**BP**	GO:0045472	response to ether	1	0.010	0.068	PPARGC1A
**BP**	GO:0070493	thrombin-activated receptor signaling pathway	1	0.010	0.068	HPGD
**BP**	GO:0097531	mast cell migration	1	0.010	0.068	CHGA
**BP**	GO:0006367	transcription initiation from RNA polymerase II promoter	2	0.010	0.068	NR3C2/PPARGC1A
**BP**	GO:0045333	cellular respiration	2	0.010	0.068	SUCLG2/PPARGC1A
**BP**	GO:0006012	galactose metabolic process	1	0.010	0.068	PPARGC1A
**BP**	GO:0032530	regulation of microvillus organization	1	0.010	0.068	PLS1

**BP**	GO:0032536	regulation of cell projection size	1	0.010	0.068	PLS1
**BP**	GO:0043201	response to leucine	1	0.010	0.068	PPARGC1A
**BP**	GO:0045820	negative regulation of glycolytic process	1	0.010	0.068	PPARGC1A
**BP**	GO:0071415	cellular response to purine-containing compound	1	0.010	0.068	PPARGC1A
**BP**	GO:0060452	positive regulation of cardiac muscle contraction	1	0.011	0.068	CHGA
**BP**	GO:1901215	negative regulation of neuron death	2	0.012	0.068	CHGA/PPARGC1A
**BP**	GO:0009812	flavonoid metabolic process	1	0.012	0.068	PPARGC1A
**BP**	GO:0014874	response to stimulus involved in regulation of muscle adaptation	1	0.012	0.068	PPARGC1A
**BP**	GO:0030812	negative regulation of nucleotide catabolic process	1	0.012	0.068	PPARGC1A
**BP**	GO:0032352	positive regulation of hormone metabolic process	1	0.012	0.068	PPARGC1A
**BP**	GO:0033604	negative regulation of catecholamine secretion	1	0.012	0.068	CHGA
**BP**	GO:0035864	response to potassium ion	1	0.012	0.068	PPARGC1A
**BP**	GO:0046321	positive regulation of fatty acid oxidation	1	0.012	0.068	PPARGC1A
**BP**	GO:0051198	negative regulation of coenzyme metabolic process	1	0.012	0.068	PPARGC1A
**BP**	GO:00075	parturition	1	0.013	0.068	HPGD

	67					
**BP**	GO:0010566	regulation of ketone biosynthetic process	1	0.013	0.068	PPARGC1A
**BP**	GO:0019372	lipoxygenase pathway	1	0.013	0.068	HPGD
**BP**	GO:0032354	response to follicle-stimulating hormone	1	0.013	0.068	PPARGC1A
**BP**	GO:0042448	progesterone metabolic process	1	0.013	0.068	PPARGC1A
**BP**	GO:0045722	positive regulation of gluconeogenesis	1	0.013	0.068	PPARGC1A
**BP**	GO:0045989	positive regulation of striated muscle contraction	1	0.013	0.068	CHGA
**BP**	GO:0055119	relaxation of cardiac muscle	1	0.013	0.068	CHGA
**BP**	GO:0071871	response to epinephrine	1	0.013	0.068	PPARGC1A
**BP**	GO:0086103	G protein-coupled receptor signaling pathway involved in heart process	1	0.013	0.068	CHGA
**BP**	GO:0097067	cellular response to thyroid hormone stimulus	1	0.013	0.068	PPARGC1A
**BP**	GO:2001170	negative regulation of ATP biosynthetic process	1	0.013	0.068	PPARGC1A
**BP**	GO:0018904	ether metabolic process	1	0.014	0.070	EPHX2
**BP**	GO:0031000	response to caffeine	1	0.014	0.070	PPARGC1A
**BP**	GO:0036270	response to diuretic	1	0.014	0.070	PPARGC1A
**BP**	GO:0	negative regulation of nucleotide	1	0.014	0.071	PPARGC1A

	030809	biosynthetic process				
**BP**	GO:0071371	cellular response to gonadotropin stimulus	1	0.014	0.071	PPARGC1A
**BP**	GO:1900372	negative regulation of purine nucleotide biosynthetic process	1	0.014	0.071	PPARGC1A
**BP**	GO:0009755	hormone-mediated signaling pathway	2	0.015	0.071	NR3C2/PPARGC1A
**BP**	GO:1902170	cellular response to reactive nitrogen species	1	0.015	0.071	PPARGC1A
**BP**	GO:0030258	lipid modification	2	0.015	0.071	EPHX2/PPARGC1A
**BP**	GO:0033002	muscle cell proliferation	2	0.015	0.071	HPGD/PPARGC1A
**BP**	GO:0019373	epoxygenase P450 pathway	1	0.016	0.071	EPHX2
**BP**	GO:0032042	mitochondrial DNA metabolic process	1	0.016	0.071	PPARGC1A
**BP**	GO:0006352	DNA-templated transcription, initiation	2	0.017	0.071	NR3C2/PPARGC1A
**BP**	GO:0071560	cellular response to transforming growth factor beta stimulus	2	0.017	0.071	HPGD/PPARGC1A
**BP**	GO:0071383	cellular response to steroid hormone stimulus	2	0.017	0.071	NR3C2/PPARGC1A
**BP**	GO:0010893	positive regulation of steroid biosynthetic process	1	0.017	0.071	PPARGC1A
**BP**	GO:0030728	ovulation	1	0.017	0.071	HPGD
**BP**	GO:0008016	regulation of heart contraction	2	0.017	0.071	FXYD3/CHGA

**BP**	GO:0071559	response to transforming growth factor beta	2	0.017	0.073	HPGD/PPARGC1A
**BP**	GO:0051195	negative regulation of cofactor metabolic process	1	0.018	0.073	PPARGC1A
**BP**	GO:0090257	regulation of muscle system process	2	0.018	0.073	CHGA/PPARGC1A
**BP**	GO:0032412	regulation of ion transmembrane transporter activity	2	0.018	0.073	FXYD3/PPARGC1A
**BP**	GO:0090140	regulation of mitochondrial fission	1	0.018	0.073	PPARGC1A
**BP**	GO:0022898	regulation of transmembrane transporter activity	2	0.019	0.074	FXYD3/PPARGC1A
**BP**	GO:0014850	response to muscle activity	1	0.019	0.074	PPARGC1A
**BP**	GO:0032528	microvillus organization	1	0.019	0.074	PLS1
**BP**	GO:0060047	heart contraction	2	0.021	0.074	FXYD3/CHGA
**BP**	GO:0006700	C21-steroid hormone biosynthetic process	1	0.021	0.074	PPARGC1A
**BP**	GO:0097066	response to thyroid hormone	1	0.021	0.074	PPARGC1A
**BP**	GO:1903579	negative regulation of ATP metabolic process	1	0.021	0.074	PPARGC1A
**BP**	GO:0032409	regulation of transporter activity	2	0.021	0.074	FXYD3/PPARGC1A
**BP**	GO:0031647	regulation of protein stability	2	0.021	0.074	TSPAN1/PPARGC1A
**BP**	GO:00159	energy derivation by oxidation of organic compounds	2	0.021	0.074	SUCLG2/PPARGC1A

	80					
**BP**	GO:0002021	response to dietary excess	1	0.021	0.074	PPARGC1A
**BP**	GO:0002026	regulation of the force of heart contraction	1	0.021	0.074	CHGA
**BP**	GO:0046885	regulation of hormone biosynthetic process	1	0.021	0.074	PPARGC1A
**BP**	GO:0051953	negative regulation of amine transport	1	0.021	0.074	CHGA
**BP**	GO:0003015	heart process	2	0.022	0.074	FXYD3/CHGA
**BP**	GO:0008209	androgen metabolic process	1	0.022	0.074	PPARGC1A
**BP**	GO:0034698	response to gonadotropin	1	0.022	0.074	PPARGC1A
**BP**	GO:0043501	skeletal muscle adaptation	1	0.022	0.074	PPARGC1A
**BP**	GO:1900543	negative regulation of purine nucleotide metabolic process	1	0.022	0.074	PPARGC1A
**BP**	GO:0045980	negative regulation of nucleotide metabolic process	1	0.023	0.074	PPARGC1A
**BP**	GO:1903522	regulation of blood circulation	2	0.023	0.074	FXYD3/CHGA
**BP**	GO:0000002	mitochondrial genome maintenance	1	0.024	0.074	PPARGC1A
**BP**	GO:0035066	positive regulation of histone acetylation	1	0.024	0.074	PPARGC1A
**BP**	GO:0045940	positive regulation of steroid metabolic process	1	0.024	0.074	PPARGC1A
**BP**	GO:0	regulation of fatty acid oxidation	1	0.024	0.074	PPARGC1A

	046320					
**BP**	GO:0061082	myeloid leukocyte cytokine production	1	0.024	0.074	CHGA
**BP**	GO:0040018	positive regulation of multicellular organism growth	1	0.025	0.074	PLS1
**BP**	GO:0043457	regulation of cellular respiration	1	0.025	0.074	PPARGC1A
**BP**	GO:0071880	adenylate cyclase-activating adrenergic receptor signaling pathway	1	0.025	0.074	CHGA
**BP**	GO:0090075	relaxation of muscle	1	0.025	0.074	CHGA
**BP**	GO:0045745	positive regulation of G protein-coupled receptor signaling pathway	1	0.025	0.075	CHGA
**BP**	GO:1901214	regulation of neuron death	2	0.025	0.075	CHGA/PPARGC1A
**BP**	GO:2000758	positive regulation of peptidyl-lysine acetylation	1	0.026	0.075	PPARGC1A
**BP**	GO:0006099	tricarboxylic acid cycle	1	0.027	0.075	SUCLG2
**BP**	GO:0071875	adrenergic receptor signaling pathway	1	0.027	0.075	CHGA
**BP**	GO:0097009	energy homeostasis	1	0.027	0.075	PPARGC1A
**BP**	GO:0000266	mitochondrial fission	1	0.028	0.075	PPARGC1A
**BP**	GO:0006101	citrate metabolic process	1	0.028	0.075	SUCLG2
**BP**	GO:0071312	cellular response to alkaloid	1	0.028	0.075	PPARGC1A

**BP**	GO:0032496	response to lipopolysaccharide	2	0.028	0.075	HPGD/PPARGC1A
**BP**	GO:0008202	steroid metabolic process	2	0.028	0.075	EPHX2/PPARGC1A
**BP**	GO:0071392	cellular response to estradiol stimulus	1	0.029	0.075	PPARGC1A
**BP**	GO:2000310	regulation of NMDA receptor activity	1	0.029	0.075	PPARGC1A
**BP**	GO:2001171	positive regulation of ATP biosynthetic process	1	0.029	0.075	PPARGC1A
**BP**	GO:0014912	negative regulation of smooth muscle cell migration	1	0.029	0.075	PPARGC1A
**BP**	GO:0045923	positive regulation of fatty acid metabolic process	1	0.029	0.075	PPARGC1A
**BP**	GO:0046839	phospholipid dephosphorylation	1	0.029	0.075	EPHX2
**BP**	GO:1904062	regulation of cation transmembrane transport	2	0.030	0.075	FXYD3/PPARGC1A
**BP**	GO:0032350	regulation of hormone metabolic process	1	0.030	0.075	PPARGC1A
**BP**	GO:0046676	negative regulation of insulin secretion	1	0.030	0.075	CHGA
**BP**	GO:0050892	intestinal absorption	1	0.030	0.075	PLS1
**BP**	GO:0071867	response to monoamine	1	0.030	0.075	PPARGC1A
**BP**	GO:0071869	response to catecholamine	1	0.030	0.075	PPARGC1A
**BP**	GO:19040	positive regulation of epithelial cell apoptotic process	1	0.030	0.075	PPARGC1A

	37					
**BP**	GO:0002237	response to molecule of bacterial origin	2	0.030	0.075	HPGD/PPARGC1A
**BP**	GO:0008207	C21-steroid hormone metabolic process	1	0.031	0.076	PPARGC1A
**BP**	GO:0072350	tricarboxylic acid metabolic process	1	0.031	0.076	SUCLG2
**BP**	GO:0070997	neuron death	2	0.031	0.076	CHGA/PPARGC1A
**BP**	GO:0045823	positive regulation of heart contraction	1	0.032	0.076	CHGA
**BP**	GO:0050832	defense response to fungus	1	0.032	0.076	CHGA
**BP**	GO:0050873	brown fat cell differentiation	1	0.032	0.077	PPARGC1A
**BP**	GO:0010677	negative regulation of cellular carbohydrate metabolic process	1	0.033	0.077	PPARGC1A
**BP**	GO:0010907	positive regulation of glucose metabolic process	1	0.033	0.077	PPARGC1A
**BP**	GO:0042181	ketone biosynthetic process	1	0.033	0.077	PPARGC1A
**BP**	GO:0006692	prostanoid metabolic process	1	0.034	0.077	HPGD
**BP**	GO:0006693	prostaglandin metabolic process	1	0.034	0.077	HPGD
**BP**	GO:0030866	cortical actin cytoskeleton organization	1	0.034	0.077	PLS1
**BP**	GO:0051602	response to electrical stimulus	1	0.034	0.077	PPARGC1A
**BP**	GO:0	adipose tissue development	1	0.034	0.077	PPARGC1A

	060612					
**BP**	GO:1901985	positive regulation of protein acetylation	1	0.034	0.077	PPARGC1A
**BP**	GO:0090278	negative regulation of peptide hormone secretion	1	0.036	0.080	CHGA
**BP**	GO:0030810	positive regulation of nucleotide biosynthetic process	1	0.036	0.080	PPARGC1A
**BP**	GO:0071354	cellular response to interleukin-6	1	0.036	0.080	PPARGC1A
**BP**	GO:1900373	positive regulation of purine nucleotide biosynthetic process	1	0.036	0.080	PPARGC1A
**BP**	GO:0043303	mast cell degranulation	1	0.037	0.080	CHGA
**BP**	GO:1903580	positive regulation of ATP metabolic process	1	0.037	0.080	PPARGC1A
**BP**	GO:0048545	response to steroid hormone	2	0.037	0.080	NR3C2/PPARGC1A
**BP**	GO:0002279	mast cell activation involved in immune response	1	0.038	0.080	CHGA
**BP**	GO:0006111	regulation of gluconeogenesis	1	0.038	0.080	PPARGC1A
**BP**	GO:0030865	cortical cytoskeleton organization	1	0.038	0.080	PLS1
**BP**	GO:0045912	negative regulation of carbohydrate metabolic process	1	0.038	0.080	PPARGC1A
**BP**	GO:0045933	positive regulation of muscle contraction	1	0.038	0.080	CHGA
**BP**	GO:0002448	mast cell mediated immunity	1	0.039	0.080	CHGA

**BP**	GO:1904707	positive regulation of vascular smooth muscle cell proliferation	1	0.039	0.080	HPGD
**BP**	GO:0002931	response to ischemia	1	0.039	0.081	PPARGC1A
**BP**	GO:0009409	response to cold	1	0.039	0.081	PPARGC1A
**BP**	GO:0070741	response to interleukin-6	1	0.039	0.081	PPARGC1A
**BP**	GO:0019369	arachidonic acid metabolic process	1	0.040	0.082	EPHX2
**BP**	GO:0006732	coenzyme metabolic process	2	0.041	0.082	SUCLG2/PPARGC1A
**BP**	GO:0009620	response to fungus	1	0.041	0.082	CHGA
**BP**	GO:2000649	regulation of sodium ion transmembrane transporter activity	1	0.041	0.082	FXYD3
**BP**	GO:0035065	regulation of histone acetylation	1	0.042	0.083	PPARGC1A
**BP**	GO:0019216	regulation of lipid metabolic process	2	0.042	0.083	EPHX2/PPARGC1A
**BP**	GO:0006636	unsaturated fatty acid biosynthetic process	1	0.043	0.084	HPGD
**BP**	GO:0010823	negative regulation of mitochondrion organization	1	0.043	0.084	PPARGC1A
**BP**	GO:0046456	icosanoid biosynthetic process	1	0.043	0.084	HPGD
**BP**	GO:1903078	positive regulation of protein localization to plasma membrane	1	0.044	0.085	PLS1
**BP**	GO:00148	striated muscle adaptation	1	0.045	0.085	PPARGC1A

	88					
**BP**	GO:0045981	positive regulation of nucleotide metabolic process	1	0.045	0.085	PPARGC1A
**BP**	GO:0071398	cellular response to fatty acid	1	0.045	0.085	PPARGC1A
**BP**	GO:1900544	positive regulation of purine nucleotide metabolic process	1	0.045	0.085	PPARGC1A
**BP**	GO:2000272	negative regulation of signaling receptor activity	1	0.045	0.085	PPARGC1A
**BP**	GO:0097755	positive regulation of blood vessel diameter	1	0.046	0.087	EPHX2
**BP**	GO:2000756	regulation of peptidyl-lysine acetylation	1	0.046	0.087	PPARGC1A
**BP**	GO:0045576	mast cell activation	1	0.047	0.087	CHGA
**BP**	GO:0030521	androgen receptor signaling pathway	1	0.048	0.087	PPARGC1A
**BP**	GO:0010676	positive regulation of cellular carbohydrate metabolic process	1	0.049	0.087	PPARGC1A
**BP**	GO:0031640	killing of cells of other organism	1	0.049	0.087	CHGA
**BP**	GO:0032922	circadian regulation of gene expression	1	0.049	0.087	PPARGC1A
**BP**	GO:0044364	disruption of cells of other organism	1	0.049	0.087	CHGA
**BP**	GO:0050433	regulation of catecholamine secretion	1	0.049	0.087	CHGA
**BP**	GO:1902305	regulation of sodium ion transmembrane transport	1	0.049	0.087	FXYD3
**BP**	GO:0	regulation of cholesterol	1	0.049	0.088	EPHX2

	090181	metabolic process				
**BP**	GO:1904377	positive regulation of protein localization to cell periphery	1	0.049	0.088	PLS1
**CC**	GO:0042589	zymogen granule membrane	1	0.009	0.119	ZG16
**CC**	GO:0042588	zymogen granule	1	0.012	0.119	ZG16
**CC**	GO:0045239	tricarboxylic acid cycle enzyme complex	1	0.012	0.119	SUCLG2
**CC**	GO:0097440	apical dendrite	1	0.015	0.119	PPARGC1A
**CC**	GO:0042629	mast cell granule	1	0.019	0.123	CHGA
**CC**	GO:0005719	nuclear euchromatin	1	0.026	0.139	PPARGC1A
**CC**	GO:0000791	euchromatin	1	0.032	0.150	PPARGC1A
**CC**	GO:0005782	peroxisomal matrix	1	0.043	0.156	EPHX2
**CC**	GO:0031907	microbody lumen	1	0.043	0.156	EPHX2
**MF**	GO:0008080	N-acetyltransferase activity	2	0.003	0.046	NAT1/NAT2
**MF**	GO:0016407	acetyltransferase activity	2	0.004	0.046	NAT1/NAT2
**MF**	GO:0016410	N-acyltransferase activity	2	0.004	0.046	NAT1/NAT2
**MF**	GO:0004955	prostaglandin receptor activity	1	0.008	0.046	HPGD

**MF**	GO:0016803	ether hydrolase activity	1	0.008	0.046	EPHX2
**MF**	GO:0004954	prostanoid receptor activity	1	0.009	0.046	HPGD
**MF**	GO:0015643	toxic substance binding	1	0.009	0.046	EPHX2
**MF**	GO:0016801	hydrolase activity, acting on ether bonds	1	0.009	0.046	EPHX2
**MF**	GO:0042577	lipid phosphatase activity	1	0.009	0.046	EPHX2
**MF**	GO:0042975	peroxisome proliferator activated receptor binding	1	0.010	0.046	PPARGC1A
**MF**	GO:0070403	NAD+ binding	1	0.012	0.048	HPGD
**MF**	GO:0004953	icosanoid receptor activity	1	0.013	0.048	HPGD
**MF**	GO:0000287	magnesium ion binding	2	0.014	0.048	SUCLG2/EPHX2
**MF**	GO:0016747	transferase activity, transferring acyl groups other than amino-acyl groups	2	0.016	0.051	NAT1/NAT2
**MF**	GO:0016922	nuclear receptor binding	1	0.017	0.051	PPARGC1A
**MF**	GO:0072509	divalent inorganic cation transmembrane transporter activity	1	0.020	0.055	SLC41A2
**MF**	GO:0016746	transferase activity, transferring acyl groups	2	0.020	0.055	NAT1/NAT2
**MF**	GO:0016878	acid-thiol ligase activity	1	0.025	0.063	SUCLG2
**MF**	GO:00011	RNA polymerase II repressing transcription factor binding	1	0.029	0.067	MIER3

	03					
**MF**	GO:0017080	sodium channel regulator activity	1	0.030	0.067	FXYD3
**MF**	GO:0043014	alpha-tubulin binding	1	0.031	0.067	PPARGC1A
**MF**	GO:0016877	ligase activity, forming carbon-sulfur bonds	1	0.033	0.069	SUCLG2
**MF**	GO:0030331	estrogen receptor binding	1	0.035	0.069	PPARGC1A
**MF**	GO:0050681	androgen receptor binding	1	0.037	0.069	PPARGC1A
**MF**	GO:1990841	promoter-specific chromatin binding	1	0.040	0.072	PPARGC1A
**MF**	GO:0003707	steroid hormone receptor activity	1	0.046	0.078	NR3C2
**MF**	GO:0051287	NAD binding	1	0.046	0.078	HPGD
